# Safety pharmacology and subchronic toxicity of jinqing granules in rats

**DOI:** 10.1186/s12917-017-1095-3

**Published:** 2017-06-17

**Authors:** Xuerong Zhou, Qian Rong, Min Xu, Yuanli Zhang, Qi Dong, Yuanling Xiao, Qiji Liu, Helin Chen, Xiaoyu Yang, Kaisheng Yu, Yinglun Li, Ling Zhao, Gang Ye, Fei Shi, Cheng Lv

**Affiliations:** College of Veterinary Medicine, Sichuan Agricultural University, Chengdu, Sichuan 611130 People’s Republic of China

**Keywords:** Jinqing granules, Rats, Toxicity, Safety pharmacology, Subchronic toxicity

## Abstract

**Background:**

Jinqing granules which are made of a mixture extract that contains Radix *Tinosporae* and *Canarii fructus* in proportions according to a longstanding formula have a good effect on the prevention and treatment of gastric ulcer disease. It has not been through safety through systematic toxicological studies, however. To provide basis for clinical application, we performed safety pharmacology and subchronic toxicity experiments in specific pathogen-free Sprague-Dawley rats.

**Results:**

In safety pharmacology experiments, Jinqing granules had no evident adverse effects on the central nervous, cardiovascular, or respiratory systems. In subchronic toxicity study, 2–8 g/kg of Jinqing granules induced no evident adverse effects on Clinical signs, body weight changes, food and water intake, death daily, indicators of urine, hematological assay, serum biochemistry, organ coefficient and histopathological examination. However, the 16 g/kg dose was associated with slightly slowed weight growth, decreased number of sperm in seminiferous tubules and increased values of serum aspartate aminotransferase and bilirubin. During the 30-day feeding test, 3 rats that received the 16 g/kg dose died, but the deaths were most likely due to trauma of oral gavage, not to drug toxicity.

**Conclusion:**

Jinqing granules given to Sprague-Dawley rats orally for 30 days at a dose of 8 g/kg or less appears safe, but higher doses were not proven safe. The significance of these observations with respect to animal usage of Jinqing granules deserves thorough investigation.

## Background

China has a several-thousand year history of using traditional medicines in clinical practice. The invention and application of traditional Chinese medications, based on specific theoretical systems and patterns of use, reflect the characteristics of Chinese culture, with its long history and rich natural resources [1]. So far, there are about 12,000 kinds of Chinese traditional medicine used in clinical, mostly herbs implied [2]. The origins and development of Chinese medicine are life’s practical experiences and medical practices of laboring people [3]. The World Health Organization [4] has reported that about 80% of the world’s populations, especially people who live in developing countries, use natural medicines to treat disease. In recent years, with rapid global economic integration, China’s pharmaceutical industry has moved strongly into the international medicine market [5]. However, the safety and effectiveness of many common Chinese traditional herbs have not been established [6, 7]. Therefore, in order to ensure the safety of prescribed traditional Chinese medicine, it is necessary that they undergo subchronic toxicity and pharmacological safety testing.

*Radix Tinosporae* is the root of *Tinospora sagittata (Oliv.)* Gagnep*,* or *Tcapillipes* Gagnep [8]*. Radix Tinosporae* is used for treating sore throat, carbuncle, furuncle, diarrhea, abdominal pain and other conditions [9–12]. *Canarii fructus,* the dry ripe fruit of *Canarium album* Raeusch, is used in the treatment of sore throat, cough and sputum viscosity [13].

Jinqing granules are made of a traditional Chinese medicine mixture extract that contains *Radix Tinosporae* and *Canarii fructus* in proportions according to a longstanding formula [14]. Jinqing granules were used in our application for a drug patent in 2014. In early trials, *Radix Tinosporae* was effective in the prevention and early treatment of *helicobacter pylori* [15]. The main active constituents of the granules are gallic acid and tetrahydropalmatine. According to literature reports on traditional Chinese medicines, *Canarium ablbum* Raeusch and *Tinospora capillipes* Ganep have favorable effects on gastric abscesses, diarrhea, and abdominal pain [8]. Even though Jinqing granules are used to treat a variety of diseases, their safety in long-term use is still questionable. About 10% of the world’s population has gastric ulcers, and about 1% of the ulcers progress to gastric cancer [16]. Therefore, Jinqing granules may have a very large market.

In early acute toxicity experiments, we found that the lethal dose 50 (LD_50_) of Jinqing granules is 5 g/kg in rats. On the basis of the LD_50_, in this work, we have studied subchronic toxicity of Jinqing granules given orally to Sprague-Dawley (SD) rats in accordance with relevant Chinese standards.

## Methods

### Jinqing granules

*Radix Tinosporae* and *Canarii fructus* were both collected from Chengdu (Sichuan, China). The plants were identified and authenticated by Professor Qiao-jia Fan of the College of Veterinary Medicine, Sichuan Agricultural University, Chengdu, China. The mixture extract that contains *Radix Tinosporae* and *Canarii fructus* in proportions according to a longstanding formula add some excipients to make Jinqing granules. The granules were completely dissolved in sterilized distilled water and made into dosages of 16 g/mL, 8 g/mL, 4 g/mL, and 2 g/mL, according to the requirements of China’s new-drug safety evaluation.

### Animals

Healthy male and female SD rats, 4 and 8 weeks old, were purchased from the specific pathogen-free facility at Chengdu Dossy Experimental Animals Co., Ltd. [License no. SCXK (Sichuan) 2008–24]. All experimental procedures involving animals were approved by Sichuan Agricultural University Animal Care and Use Committee (registration No. SCAU2016061801). Based on the principles of the International Committee on Laboratory Animals, the rats were separated equally according to gender and maintained at 20–25 °C and relative humidity of 55 ± 5%. Artificial lighting was adjusted to 12 h of light (from 08:00 to 20:00) and 12 h of darkness from 20:00 to 08:00. Conventional laboratory feed was provided by the Chengdu Dashuo Biology Science and Technology Co., Ltd. The rats were allowed to drink water freely, and they acclimated for 7 days before experiments were begun.

### Safety pharmacology experiments

Safety pharmacology experiments were performed according to guidelines of the Veterinary Drugs Control [17–20].

Fifty SD rats were randomly distributed into 5 groups (5 female and 5 male in each group). The five groups were: Group I, normal control group (sterile distilled water); Group II, 2 g/kg Jinqing granules; Group III, 4 g/kg Jinqing granules; Group IV, 8 g/kg Jinqing granules; and Group V, 16 g/kg Jinging granules. Animals were treated daily at 9 a.m. with gastric infusion in a volume of 10 ml/kg given for 7 days.

We observed the animals for behavior, posture, salivation, pupil change, muscle tremors, hair, and feces were observed from 10 a.m. to 16 p.m. continually for 14 days [17–20]. In order to prevent living animals from eating animals that had died due to accumulation of drug in the body, endangered or dead rats were autopsied promptly [21].

Before and 24 h after the last administration of control water or Jinqing granules, the rats were placed in the versatile recorder of rats’ locomotor activity and allowed to adapt to the environment for 30 min. Their activity (number of movements in 1 min) was recorded by watching the rats’ movements in order to determine if this aspect of the central nervous system was normal.

The climbing pole test was performed in order to evaluate the mobility of the rats after receiving the medication for various times. Twenty-four hours after the last administration, a smooth metal bar was erected vertically and fixed at the bottom. The rats were placed at the top of the metal bar and allowed to descend head first. Their ability to coordinate this activity was graded as: Level 0, normal step-by-step climbing down; Level 1, coasting down; Level 2, inability to hang from the lever; Level 3, discoordination over a period of time.

On the day before medication was given, the day after it was given, and the eighth day after being given, 3% pentobarbital sodium was injected intraperitoneally to anesthetize the rats [17–20]. The animals’ heart rate, electrocardiogram, and the depth of breathing and respiratory rate were recorded with the BL-420F multi-channel physiological signal acquisition processing system.

### Subchronic toxicity experiment

The toxicity experiments were conducted according to the Economic Cooperation and Development Guideline 407 [22].

One hundred-twenty rats were randomly distributed into 6 groups (10 female and 10 male rats in each group). The 6 groups were: Group I, normal group; Group II, saline control group; Group III, 2 g/kg; Group IV, 4 g/kg; Group V, 8 g/kg; Group VI, 16 g/kg in a volume of 10 ml/kg Jinging granules. Animals were treated daily at 9 a.m. with gastric infusion in a volume of 10 ml/kg given for 30 days. The determination of repeated dose 30-day oral toxicity was carried out according to the Organization for Economic Cooperation and Development Guideline 407 [22]. In order to avoid accumulation of drug that existed in the dead rats in the living body by eating, and analyzed the causes of death in rats, the dead rats were needed to anatomical analysis immediately. [21].

Daily from 10 a.m. to 16 p.m., we observed the rats’ behavior, attitude, presence of saliva, pupillary change, muscle tremors, hair texture, food intake, water intake, feces, poisoning, and death, continually for 30 days. Weight was recorded every three days.

At the end of medication period, urine samples were collected from all animals. The samples were sent to Chengdu Lilai Biology Science and Technology Co., Ltd., for determination of pH, ascorbic acid, specific gravity, glucose, leukocyte counts, nitrite, protein, ketones, urobilinogen, and bilirubin.

About 2.0 mL of blood was collected in an anti-coagulation tube containing sodium citrate to measured hematologic indices, and another 2.0 mL blood was collected in non-heparinized tubes to measured serum biochemistry.

Rats were sacrificed after blood was collected and were opened surgically for evaluation of possible pathological changes. The weights of liver, heart, spleen, lung, kidney, brain, ovaries, and testes were recorded. The relative weight of organs was calculated according to the formula: organ coefficient = organ weight / body weight ×100%) [23, 24].

Organs were soaked in 10% solution of buffered formalin (pH 7.4) for three days, followed by dehydration, embedding, sectioning, dewaxing, and staining with hematoxylin-eosin. Pathologic alterations were assessed with a Nikon 80i optical microscope. Photomicrographs were taken for reference.

### Statistical analysis

Results were analyzed with SPSS 19.0 statistical software. The mean and standard deviation were expressed. One-way analysis of variance (ANOVA) and Newman-Keuls post hoc test were performed for statistical analyses. Statistically significant difference was defined as *p* < 0.05.

## Results

### Safety pharmacology experiments

During the safety pharmacology experiments, no deaths occurred, and among all animals in all groups, no abnormalities in fur, posture, pupils, feces, salivation, or muscle tremors were noted. Each dose-group of rats was no evident abnormalities of behavior controlled by the central nervous system, e.g., posture, change in pupil size, salivation, gait, and muscle tremors.

The index for active movement in each dose group of animals receiving Jinqing granules revealed no differences from the index of the control group (*P* > 0.05). Thus, Jinqing granules had no evident effect on the rats’ spontaneous activity. The results are shown in Table 1.

**Table 1:** Jinqing granules effect on autonomic activities in rats (*n* = 10)

Jinqing dose	Gender	Before dosing (times)	After dosing (times)
Control group	Male	160.00 ± 23.97	183.00 ± 11.18
	Female	175.00 ± 20.49	161.43 ± 17.82
2 g/kg	Male	153.00 ± 12.75	138.00 ± 52.99
	Female	168.00 ± 12.55	182.00 ± 27.29
4 g/kg	Male	145.00 ± 55.69	125.00 ± 63.54
	Female	161.00 ± 9.62	135.00 ± 33.73
8 g/kg	Male	179.00 ± 28.95	167.00 ± 54.04
	Female	156.00 ± 11.40	136.00 ± 23.82
16 g/kg	Male	170.00 ± 14.72	155.00 ± 39.37
	Female	178.75 ± 59.35	176.25 ± 14.36

Values represent means ± SD (*N* = 10). *P* values for all comparisons of before dosing and after dosing were ≥0.05.

In the climbing pole test, the treatment groups and the control-group rats both climbed down step-by-step, i.e., at level 0, which is evidence that Jinqing granules had no discernable effect on the animals’ coordination.

**Fig. 1:**
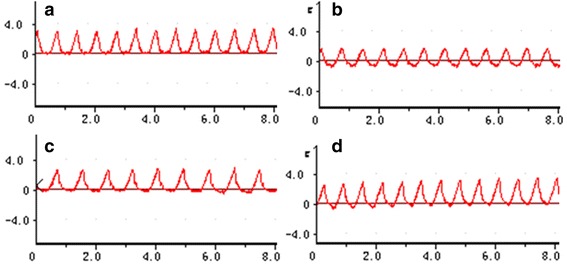
The effect of Jinqing granules on breath rate of rats. **a** Control group; **b** The Group V before administration; **c** The Group V at the end of the second administration; **d** The Group V at a week after the last administration

**Fig. 2:**
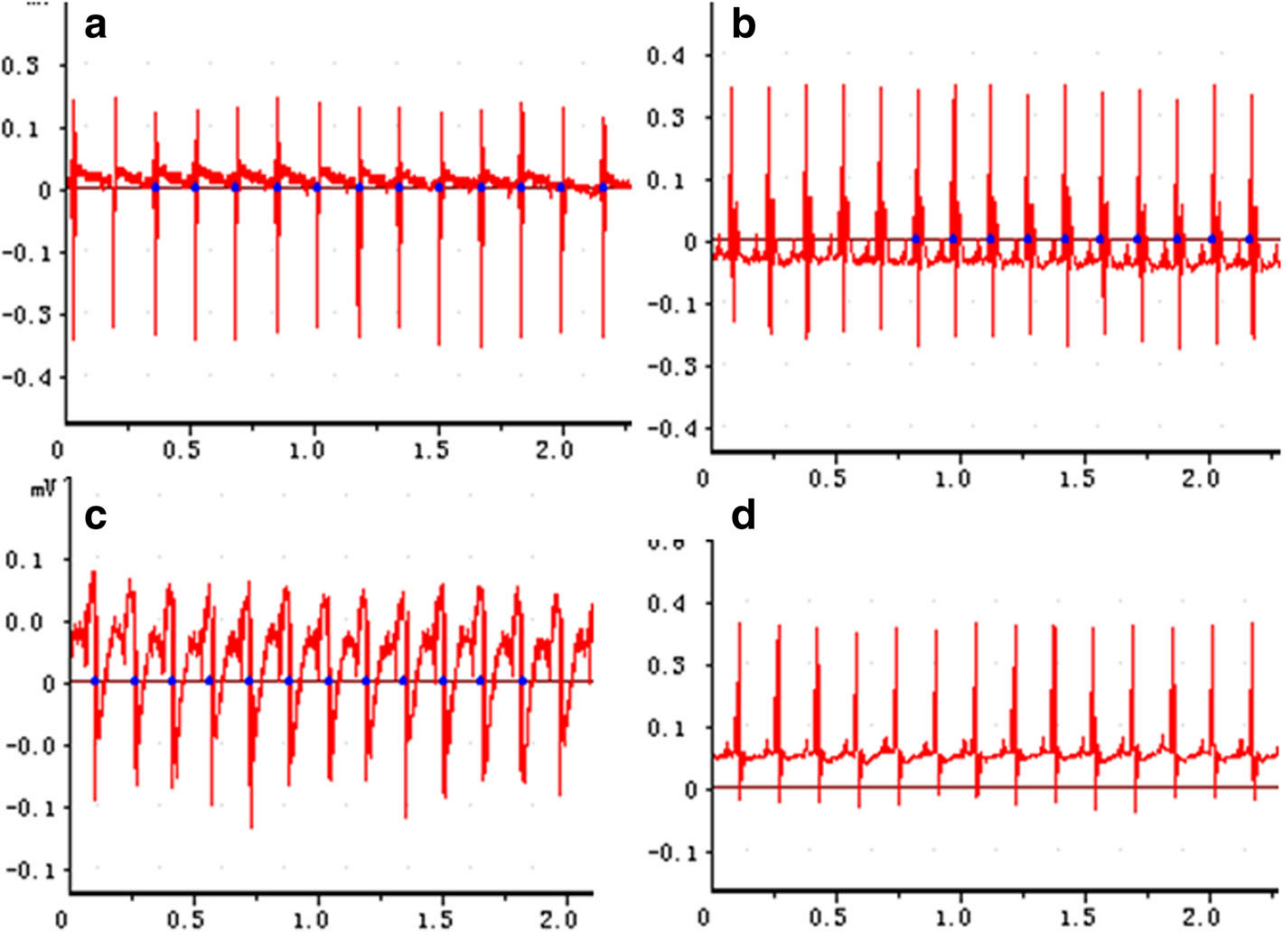
The effect of Jinqing granules on heart rate of rats. **a** Control group; **b** The Group V before administration; **c** The Group V at the end of the second administration; **d** The Group V at a week after the last administration

The measurements of heart rate and respiratory rate on the day before administration of the medication, the day after administration, and the eighth day after administration revealed no significant differences between control-group rats and those who received Jinqing granules at any dose (*P* > 0.05). At the same time, at three different time points within the same group, there were no significant differences in heart rate and respiratory rate (*P* > 0.05). Thus, we conclude that Jinqing granules had no discernable significant effects on the cardiovascular and respiratory systems of the SD rats. The results are listed in Tables 2 and 3. Figure 1 illustrates time-breath curves of Jinqing granules’ effect on the rats and Fig. 2 is time-heart curves.

**Table 2:** The effects of Jinqing granules on the heart rate of rats (*n* = 10)

Jinqing dose	Gender	Before dosing	The second day after dosing	One week after dosing
Control group	Male	333.60 ± 25.96	372.40 ± 27.17	397.00 ± 36.92
	Female	387.60 ± 10.21	358.80 ± 70.38	406.40 ± 12.90
2 g/kg	Male	328.20 ± 23.30	369.40 ± 48.82	396.20 ± 10.64
	Female	392.20 ± 9.39	420.40 ± 25.31	342.60 ± 24.33
4 g/kg	Male	330.20 ± 28.96	336.40 ± 38.40	369.20 ± 57.80
	Female	389.20 ± 11.61	324.00 ± 16.55	382.20 ± 49.47
8 g/kg	Male	333.60 ± 25.96	308.60 ± 35.81	388.60 ± 35.41
	Female	387.60 ± 10.21	341.60 ± 48.69	362.00 ± 41.41
16 g/kg	Male	334.50 ± 21.89	371.25 ± 27.37	388.50 ± 13.96
	Female	372.25 ± 10.84	403.25 ± 74.22	367.25 ± 34.88

Values represent means ± SD (*N* = 10). *P* values for all comparisons of before dosing and after dosing were ≥0.05

**Table 3:** The effects of Jinqing granules on the respiratory rate of rats (*n* = 10)

Jinqing dose	Gender	Before dosing	The second day after dosing	One week after dosing
Control group	Male	96.00 ± 11.22	81.20 ± 11.63	91.80 ± 14.17
	Female	88.80 ± 12.30	94.20 ± 23.88	105.80 ± 29.45
2 g/kg	Male	96.00 ± 14.70	86.40 ± 13.89	93.80 ± 6.94
	Female	92.40 ± 15.06	80.00 ± 17.39	113.80 ± 5.76
4 g/kg	Male	96.80 ± 12.30	77.00 ± 9.43	84.80 ± 7.60
	Female	94.25 ± 13.01	74.40 ± 10.55	97.60 ± 17.42
8 g/kg	Male	91.20 ± 9.86	70.80 ± 9.18	94.20 ± 15.06
	Female	88.80 ± 12.30	68.20 ± 22.65	98.60 ± 15.55
16 g/kg	Male	96.00 ± 11.22	71.59 ± 13.30	80.00 ± 7.48
	Female	95.34 ± 14.70	87.75 ± 4.5	112.25 ± 22.40

Values represent means ± SD (*N* = 10). *P* values for all comparisons of before dosing and after dosing were ≥0.05

### Subchronic toxicity experiments

During the 30-day feeding test, 2 male and 1 female rats in Group VI died. Autopsy of these animals disclosed findings suggestive of improper oral lavage administration; no signs of Jinqing toxicity were found. Also, among the various groups of animals, no abnormalities were seen in fur, action, posture, pupillary change, diet, salivation, muscle tremors, and feces.

The rats’ food and water consumption are shown in Table 4. Water consumption was not significantly different between groups that received Jinqing granules and those in the control group (*P* > 0.05). Food intake also was similar in the groups that received Jinqing granules and the control group, except that animals, both male and female, that had received 16 g/kg (Group VI) consumed slightly less food (*P* < 0.05).

**Table 4:** Mean food and water consumption of Sprague-Dawley rats orally administered Jinqing granules for 30 days (*n* = 20)

Jinqing dose	Gender	Diet (g/rat/day)	Water (ml/rat/day)
Control group	Male	20.5	31.7
	Female	20.1	31.4
Saline group	Male	21.0	31.6
	Female	20.7	31.8
2 g/kg	Male	20.3	31.3
	Female	20.2	31.0
4 g/kg	Male	20.1	30.9
	Female	19.8	31.5
8 g/kg	Male	19.5	31.2
	Female	19.4	30.6
16 g/kg	Male	17.6^*^	30.7
	Female	17.3^*^	30.3

*P < 0.05 significant difference from control

**Fig. 3:**
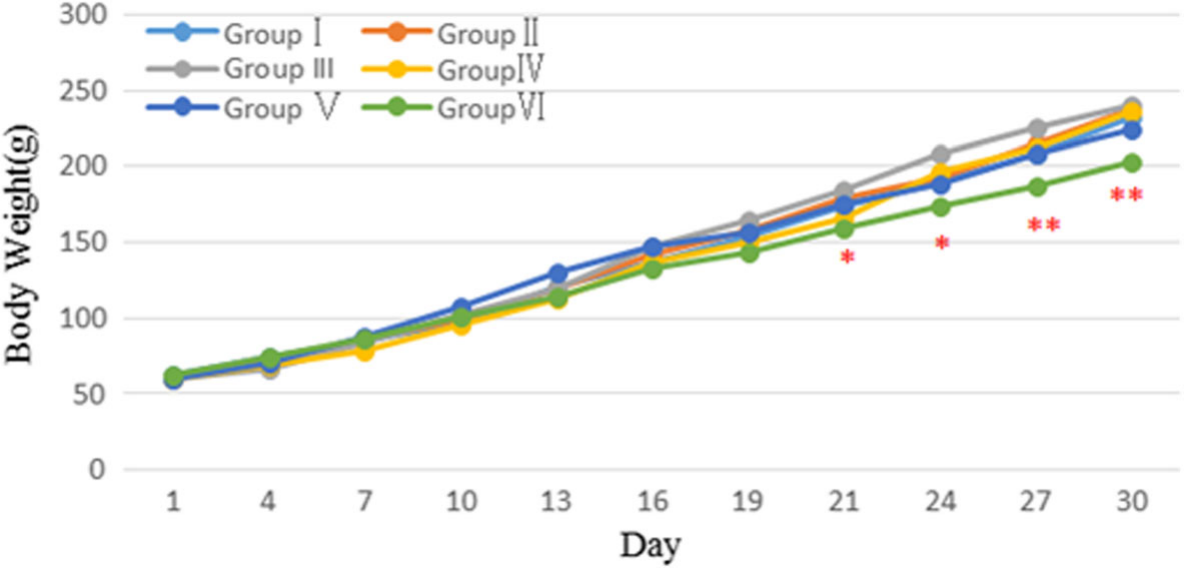
Body weight changes of male rats during the sub-chronic toxicity study. Data are presented as the mean ± SD (5 rats/sex/group); *p < 0.05, **p < 0.01 Group VI vs. the Group I

**Fig. 4:**
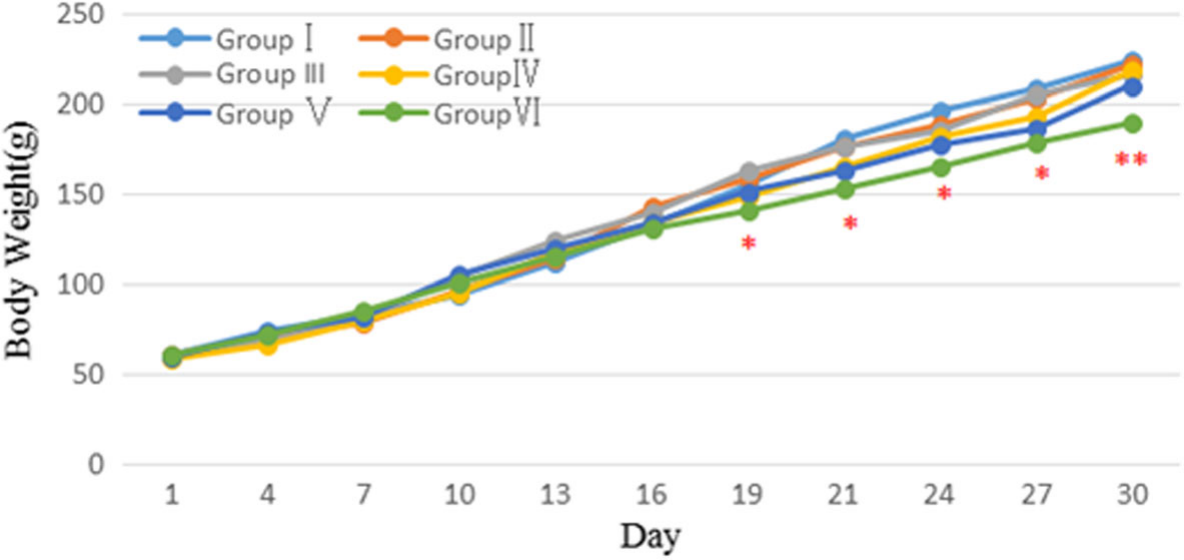
Body weight changes of female rats during the sub-chronic toxicity study. Data are presented as the mean ± SD (5 rats/sex/group); *p < 0.05, **p < 0.01 Group VI vs. the Group I

Figure 3 illustrates the weight gain curves of the various groups of male rats, and Fig. 4 is female rats. No difference was recorded between the control group (Group I) and the saline group (Group II) (*P* > 0.05). The body weight gain of Groups III -V (2 g/kg to 8 g/kg Jinqing granules) also were not significantly different from the body weight gain of control animals (*P* > 0.05). However, the weight gain of Group VI (16 g/kg) male rats compared with weight gain of control rats was slightly less. Group VI female rats had a similar decline in weight gain, from the 19th to the 31st day.

Indicators of urine samples that, the ascorbic acid in the negative rate is up to 90%. Therefore, other indicators of false negative rate can be ignored. The values for pH, specific gravity, glucose, white blood cells, and nitrite were normal in all animals at all doses of Jinqing granules. The values for bilirubin was slightly increased in the 16 g/kg dose compared to control values (*p* < 0.05). The values of occult blood, protein, ketone, and urobilinogen were slightly increased but did not reach statistical significance (*P* > 0.05). Dataes is in Table 5.

**Table 5:** Effect of Jinqing granules on indicators of urine in rats (*n* = 20)

Parameters	GLU%				WBC%				NIT%			
	(−)	(+)	(++)	(+++)	(−)	(+)	(++)	(+++)	(−)	(+)	(++)	(+++)
Control group	100				100				100			
Saline group	100				100				100			
2 g/kg	100				100				100			
4 g/kg	100				100				100			
8 g/kg	100				100				100			
16 g/kg	100				100				100			
Control group	100				100				95	5		
Saline group	95		5		95		5		100			
2 g/kg	95	5			100				100			
4 g/kg	100				100				100			
8 g/kg	100				100				100			
16 g/kg	85	5	5	5	90	5	5		85	10		5
Control group	95	5			95		5		95	5		
Saline group	90	10			100				100			
2 g/kg	95			5	100				100			
4 g/kg	90	5		5	95	5			95	5		
8 g/kg	90		10		100				95	5		
16 g/kg	80*	10	5	5	85	10	5		100			

*P < 0.05 significant difference from control

*GLU* glucose, *WBC* white blood cells, *NIT* nitrite, *PRO* protein, *BLD* occult blood, *KET* ketone, *BIL* bilirubin, *URO* urobilinogen, *V*_*C*_ ascorbic acid

Table 6 presents the hematologic indices in the various groups of rats. The indices were not significantly different from control values at any dose of Jinqing granules, except for a slightly higher lymphocyte count at the 16 g/kg dose. Although some values in Groups III - VI were higher than in the control group, the differences were not statistically significant.

**Table 6:** Effect of subchronic administration of Jinqing granules on hematologic indices in rats (*n* = 20)

Parameters	WBC (×10^9^/L)	RBC (×10^12^/L)	PLT (×10^9^/L)	HGB (g/L)	LYM% (×10^9^/L)	GRAN (×10^9^/L)	HCT %
Control group	7.82 ± 1.12	8.52 ± 0.82	1024.67 ± 141.96	159.80 ± 8.56	78.28 ± 3.70	1.71 ± 0.40	35.83 ± 3.18
Saline group	8.58 ± 0.75	9.14 ± 0.88	1313.29 ± 105.33	166.86 ± 8.73	80.75 ± 4.43	1.93 ± 0.41	36.01 ± 2.22
2 g/kg	7.59 ± 1.54	8.17 ± 0.72	1140.13 ± 266.16	157.87 ± 14.27	79.11 ± 3.41	1.89 ± 0.28	38.61 ± 4.47
4 g/kg	7.91 ± 1.17	8.56 ± 2.04	1105.07 ± 115.30	152.53 ± 8.63	81.75 ± 0.96	1.52 ± 0.27	37.59 ± 3.75
8 g/kg	7.93 ± 1.94	8.73 ± 1.02	1076.13 ± 98.33	155.67 ± 7.89	76.99 ± 1.78	1.57 ± 0.46	36.15 ± 4.50
16 g/kg	7.32 ± 2.07	9.19 ± 0.49	1297.10 ± 86.70	167.4 ± 12.15	82.07 ± 2.97^*^	1.82 ± 0.42	38.96 ± 1.48

Values represent mean ± SD

*WBC* white blood cell, *RBC* red blood cell, *PLT* blood platelet, *HGB* hemoglobin, *LYM* lymphocytes, *Gran* neutrophils, *HCT* red blood cell specific volume

*P < 0.05, significantly different from control values

Serum biochemistry values of treated animals and controls are presented in Table 7. The only statistically significant differences in values between animals that received Jinqing granules and control animals was an increase in aspartate aminotransferase and total bilirubin at the 16 g/kg dose (*P* < 0.05).

**Table 7:** Effect of subchronic administration of Jinqing granules on serum biochemistry measurements in rats (*n* = 20)

Parameters	ALT(U/L)	AST(U/L)	TP(g/L)	TG(g/L)	GLU(mmol/L)	UREA(mmol/L)	CREA (mmol/L)	T-Bil(g/L)	ALB(g/L)
Control group	32.17 ± 5.59	105.58 ± 31.84	57.59 ± 4.12	0.34 ± 0.17	5.33 ± 1.77	5.64 ± 1.18	32.76 ± 3.95	2.32 ± 0.43	25.81 ± 4.46
Saline group	34.13 ± 3.90	111.45 ± 22.26	55.33 ± 5.57	0.43 ± 0.15	5.15 ± 2.67	5.51 ± 1.36	33.94 ± 6.81	2.36 ± 0.33	26.38 ± 4.14
2 g/kg	31.63 ± 3.84	119.03 ± 31.76	56.16 ± 3.64	0.32 ± 0.08	5.46 ± 1.77	5.66 ± 0.88	29.55 ± 8.76	2.87 ± 0.27	26.76 ± 4.67
4 g/kg	35.72 ± 5.99	119.78 ± 15.37	59.07 ± 5.52	0.33 ± 0.21	5.19 ± 1.64	5.46 ± 0.96	31.74 ± 7.14	2.44 ± 0.41	27.29 ± 5.33
8 g/kg	33.96 ± 3.97	125.76 ± 20.33	55.98 ± 3.73	0.34 ± 0.07	5.04 ± 1.44	6.02 ± 0.66	35.28 ± 6.94	3.15 ± 0.35	25.91 ± 4.58
16 g/kg	37.69 ± 3.03	137.63 ± 26.44^*^	58.45 ± 4.09	0.45 ± 0.09	4.88 ± 2.31	6.23 ± 1.07	36.66 ± 5.46	3.43 ± 0.42^*^	27.61 ± 8.61

Values represent means ± SD

*ALT* alanine aminotransferase, *AST* aspartate aminotransferase, *TP* total protein, *TG* triglycerides, *GLU* glucose, *UREA* blood urea nitrogen, *CREA* creatinine, *TC* total cholesterol, *ALB* albumin

*P < 0.05, significantly difference from control values

Organ coefficients are presented in Table 8. Slightly higher values in the liver and ovaries of animals treated with 16 g/kg of Jinqing granules than in control animals were recorded (*p* < 0.05). Otherwise, no differences among the groups were found.

**Table 8:** Effect of subchronic administration of Jinqing granules on the organ coefficient (g/ g*100%) of male and female rats (*n* = 20)

Parameters	gender	Heart	Liver	Spleen	Lung	Kidney	Testis or Ovary
Control group	Male	0.35 ± 0.01	3.24 ± .024	0.30 ± 0.04	0.51 ± 0.05	0.79 ± 0.06	1.25 ± 0.12
	Female	0.37 ± 0.05	3.26 ± 0.19	0.31 ± 0.03	0.47 ± 0.02	0.76 ± 0.06	0.05 ± 0.01
saline group	Male	0.37 ± 0.03	3.36 ± 0.10	0.28 ± 0.04	0.53 ± 0.05	0.80 ± 0.04	1.27 ± 0.10
	Female	0.39 ± 0.03	3.45 ± 0.14	0.29 ± 0.03	0.52 ± 0.02	0.77 ± 0.05	0.04 ± 0.01
2 g/kg	Male	0.35 ± 0.02	3.37 ± 0.19	0.32 ± 0.05	0.54 ± 0.06	0.78 ± 0.04	1.24 ± 0.14
	Female	0.37 ± 0.04	3.37 ± 0.36	0.27 ± 0.06	0.55 ± 0.08	0.81 ± 0.11	0.05 ± 0.01
4 g/kg	Male	0.35 ± 0.01	3.39 ± 0.24	0.31 ± 0.05	0.52 ± 0.04	0.75 ± 0.06	1.21 ± 0.10
	Female	0.36 ± 0.02	3.27 ± 0.22	0.31 ± 0.07	0.50 ± 0.05	0.79 ± 0.04	0.05 ± 0.01
8 g/kg	Male	0.36 ± 0.04	3.44 ± 0.24	0.28 ± 0.04	0.54 ± 0.05	0.78 ± 0.06	1.28 ± 0.11
	Female	0.35 ± 0.02	3.32 ± 0.18	0.30 ± 0.03	0.52 ± 0.05	0.76 ± 0.02	0.05 ± 0.01
16 g/kg	Male	0.34 ± 0.05	3.49 ± 0.37^*^	0.26 ± 0.05	0.54 ± 0.13	0.83 ± 0.11	1.41 ± 0.13^*^
	Female	0.36 ± 0.03	3.52 ± 0.16^*^	0.26 ± 0.05	0.54 ± 0.04	0.80 ± 0.06	0.06 ± 0.01

Values represent means ± SD

*P < 0.05; sig.nificantly different from control

**Fig. 5:**
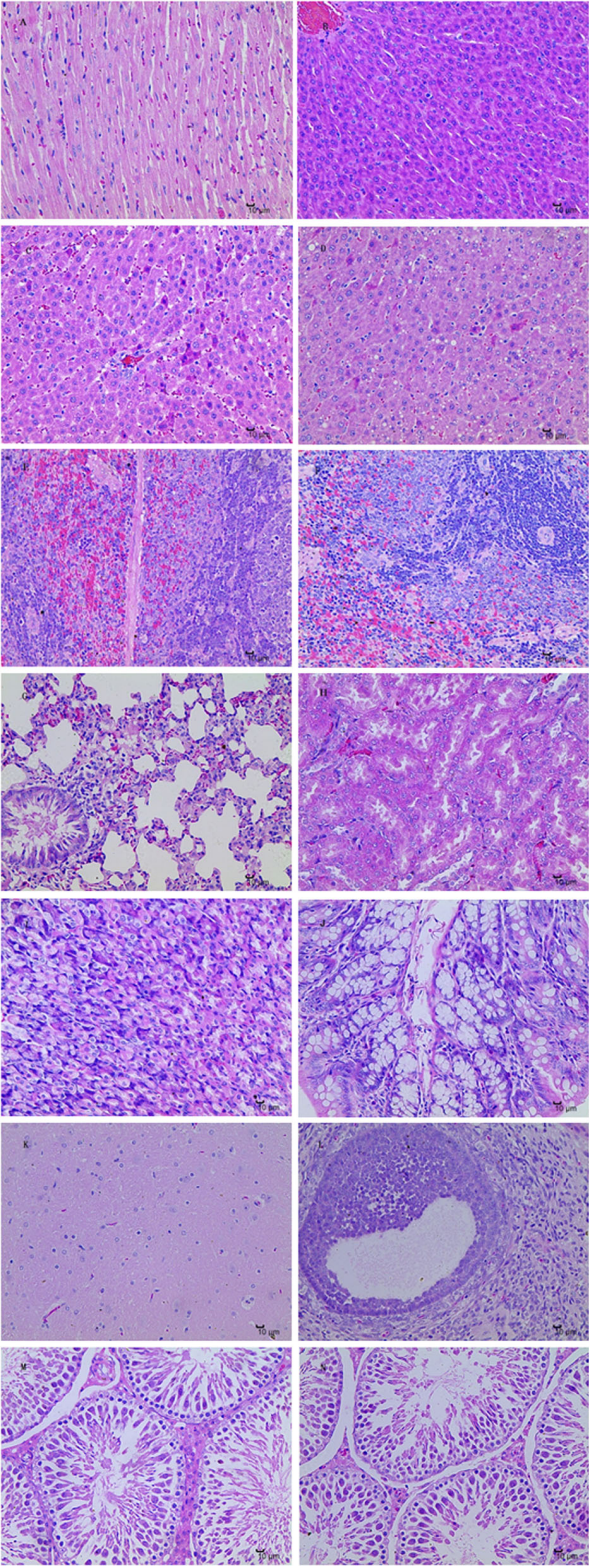
H&E staining of the liver, kidney, brain, heart, lung, spleen from the rats used in the subchronic toxicity test. *N* = 3 per group, scale bar = 10 μm; 400×. **a** Representative section of heart tissue (Group I- VI), with no histological abnormality. **b** Liver, 0 g/kg, Group I, without histological abnormality. **c** Liver, Group VI animals, with expansion of liver sinusoids, **d** Liver, Group VI animals, with various degrees of vacuolar degeneration in hepatocytes. **e** Spleen, 0 g/kg (Group I), with no histological abnormality. **f** Spleen, Group VI rats, with decreased numbers of lymphocytes and increased numbers of macrophages. **g** Representative section of Lung, Group I-VI, with no histological abnormality. **h** Representative section of kidney, Group I-VI, with no histological abnormality. **i** Representative section of stomach, Group I-VI, with no histological abnormality. **j** Representative section of intestines, Group I-VI with not histological abnormality. **k** Representative section of brain, Group I-VI, with no histological abnormality. **l** Representative section of ovary, Group I- VI, with no histological abnormality. **m** Representative sections of testicle, Group I-V, with testicular tubules were arranged neatly and tightly. **n** Testicle, Group VI, with the number of sperm in seminiferous tubules appeared decreased

After the 30-day trial, the heart, liver, spleen, lung, kidney, stomach, intestines, brain, testis and ovary were taken from 3 rats of each group for histopathologic examination (Fig. 5). Tissue damage was seen only in animals that had received the 16 g/kg dose of Jinqing granules. In the heart, each group had a complete tegument and ventricular and ventricular muscle contour (Fig 5a), with no evident pathological damage. In the liver, compared with the control group (Fig 5b), Group VI animals had expansion of liver sinusoids (Fig 5c), and various degrees of vacuolar degeneration in hepatocytes (Fig 5d). In the spleen, in control animals (Group I), the capsule was intact, the boundary between white pulp and red pulp was distinct, and the lymphocyte number was normal (Fig 5e). In contrast, in Group VI rats, the lymphocyte were decreased, and the number of macrophages was increased (Fig 5f). In the lung, the capsules of all groups were complete, and all levels of bronchi (primary bronchus, secondary bronchi, and tertiary bronchi) were neatly arranged in all animals (Fig 5g). In the kidney, pathological changes were not evident in any group. The renal capsule was intact; the boundary between cortex and medulla was clear; and the renal tubules and collecting ducts were arranged neatly, with normal density (Fig 5h). In the stomach, in Group I to Group VI, structural integrity of the simple columnar epithelium, muscularis mucosa and muscularis propria were maintained (Fig 5i). In the intestines, the mucosa, submucosa, muscular layer, and outer membrane layer structure were intact, and boundaries between these structures were distinct in all animal groups (Fig 5j). In brain, the nerve fiber layer, cerebral cortex, and hippocampus pyramidal cells were arranged neatly and tightly, without inflammatory infiltration or hemorrhagic foci (Fig 5k). In the ovary, from Group I to Group VI, the morphology and quantity of ovarian primordial follicles, proliferating follicles and mature follicles were normal compared with those features in the control animals (Fig 5l). In the testes, seminiferous tubules had a complete structure, and the spermatogenic cells resting in testicular tubules were arranged neatly and tightly (Fig 5m); however, compared with the control group, the number of sperm in seminiferous tubules appeared decreased in Group VI animals (Fig 5n).

## Discussion

In this work, we conducted subchronic toxicity tests [20] and safety pharmacology experiments [17–20] in rats treated with Jinqing granules in order to assess the potential toxicity of this traditional Chinese medicine. Conventional laboratory food was chosen in order to have a uniform diet without variation throughout the testing period.

Safety pharmacology is a pharmacological method used to study drugs given at the recommended treatment doses, or higher doses, which may exert harmful pharmacological effects or adverse drug reactions [25–27]. Safety pharmacology research can provide information for clinical research and safe drug use, and can be used for long-term toxicity testing as a reference for the design and development of new indications [28–30]. The present research, conducted according to safety pharmacology research technical guidelines for natural medicines, tested the effects of Jinqing granules on critical body functions, such as respiratory rate, heart rate, and central nervous system activities.

Because the central nervous system plays a vital role in the development of and the regulation of physiological behaviors, the rats’ daily routine activities and the climbing pole test were used to detect effects of Jinqing granules on the central nervous system [31]. Treatment at all doses did not affect the animals’ performance in these rudimentary tests. Although the possibility of subtle central nervous system effects was not excluded, the tests gave no evidence of gross damage.

Measurement of rats’ heart rate also did not reveal an abnormal effect in the Jinqing-treated animals, which is some evidence that the drug may have no adverse effect on the cardiovascular system [32, 33].

The subchronic toxicity testing is used to assess the long-term safety of exogenous substances [34]. In our 30-day clinical trial, two male and one female rats in Group VI died. Because autopsy of these animals disclosed signs of damage from improper oral lavage, and no other abnormalities were found at autopsy, we feel that toxic effects of Jinqing were not the cause of the deaths.

Changes in body weight can be used as indicators of general toxicity of drugs and chemicals [35–37]. In our study, Jinqing granules had only minimal effect on the rate of weight gain, and the effect was only statistically significant at the highest dose (Group VI; 16 g/kg) and not until 3 to 4 weeks of treatment. Whether this effect was due to effects of the medication is unknown.

Hematological analyses in humans and animals are a sensitive indicator of drug and chemical toxicity [38–40]. In analyses of blood indices, we found no abnormalities in several measurements in the Jinqing-treated animals (white blood cell count, red blood cell count, platelet count, hemoglobin, and neutrophil count), but a slight, statistically significant increase in lymphocyte counts (from 77 to 82 × 10 ^9^/ml in Group IV animals) was found; we suspect this difference is of no clinical significance, because they were within normal physiological ranges [41–43].

As with other measurements, the biochemical tests revealed little abnormality in the Jinqing-treated animals, although aspartate aminotransferase and total bilirubin were modestly increased in Group VI animals. Aspartate aminotransferase is released into the blood [44], when liver-cell injury occurs, but an increase in serum aspartate aminotransferase value is not specific for liver disease [45]. Bilirubin is a breakdown product of normal heme catabolism [46]. With disease of the liver or biliary system, serum bilirubin values may rise [47], but increased values may be present in hematologic or other diseases as well. In our study, the combination of increased serum aspartate aminotransferase and total bilirubin raise the possibility of liver injury, but whether the mild abnormalities seen only with the highest doses of Jinqing are of clinical significance is unknown.

Serum creatinine is an important index of kidney function [48, 49], and blood urea nitrogen values can reflect glomerular filtration function [50]. That these tests were normal in our rats is evidence that Jinqing granules, even at high doses, did not adversely affect renal function. This conclusion is supported also by the normal urinary pH, specific gravity, and protein values in treated animals.

Organ coefficient is an important index in the evaluation of new-drug preclinical toxicity and effect on target organs [51, 52]. Studies have reported that the organ coefficient may be increased due to congestion, edema, hypertrophy, and other causes, and it may be decreased due to organ shrinking and degenerative changes [53, 54]. These effects may not be directly attributed to a direct effect of the drug being tested, and could be due a secondary change. In this study, no abnormalities in organ coefficient were found, except for slight but statistically significantly decreased values for the liver and testes at the 16 g/kg dose.

After the 30-day trial, histological examination of the heart, liver, spleen, lung, kidney, stomach, intestines, brain, testes and ovary found minimal changes, except for decreased number of sperm in seminiferous tubules of Group VI animals. Therefore, an adverse effect of Jinqing on reproductive capability and hepatotoxicity have to be considered.

Brain is a crucial organ for regulating mental development, and promotes endocrine release of human [55, 56]. The hippocampus and cortex play an absolutely essential role in learning and memory [57, 58]. Hypothalamus regulates a variety of functions, including sleep, body temperature, thirst and hunger. In this study, histopathological displayed a normal neuronal structure in the hippocampus, cortex and hypothalamus, without inflammatory infiltrate or hemorrhagic lesions. In addition, in the subchronic toxicity test the change of behavior was not observed. Tests have shown that Jinqing granules had no effect on rats’ brain.

We acknowledge that our study has limitations. The most consequential may be the relatively short duration of Jinqing feeding; trials of longer-term administration of the medication are needed. Also, results of tests in Sprague-Dawley rats may not be applicable to other animal species or to humans. Finally, despite our extensive testing for adverse events associated with Jinqing feeding, the tests may not have been sufficiently sensitive or specific to detect all adverse events.

The doses of Jinquing granules we fed to rats are well above those usually used in the treatment or prevention of gastric ulcers. Thus, although our findings are not directly transferrable to porcine husbandry, they give some assurance about the safety of Jinqing feeding.

## Conclusions

Extensive safety pharmacology and subchronic toxicity tests on rats fed ultra-high doses of Jinqing granules (up to 16 g/kg) revealed little evidence of toxicity on the animals’ nervous, cardiovascular, respiratory, renal, and hematological systems. However, the highest dose was associated with slightly slowed weight growth and minor histologic changes of unknown significance, decreased numbers of sperm in seminiferous tubules, and increased values of serum aspartate aminotransferase and bilirubin. During the 30-day feeding test, 3 rats that received the 16 g/kg dose died, but the deaths were most likely due to trauma of oral gavage, not drug toxicity. Jinqing granules given to Sprague-Dawley rats orally for 30 days at a dose of 8 g/kg or less appears safe, but higher doses were not proven safe. The significance of these observations with respect to usage of Jinqing in animals, or possibly in humans, deserves thorough investigation.
